# Treatment of Typhoid Fever

**Published:** 1893-02-18

**Authors:** 


### ROYAL SOUTHERN HOSPITAL, LIVERPOOL.

### TREATMENT OF TYPHOID FEVER.

Typhoid fever is the disease above all others, in which we see the beneficial effects of sound and proper treatment, not so much the medicinal as the dietetic treatment. Who has not seen the terrible, nay, even fatal, effects of indiscretion in diet during the course of this disease, a small piece of bread, or an orange, either brought in by friends or given them by a fellow patient, causing a relapse, and if not causing perforation, prolonging the disease by many weeks. The temperature illustrates this very well. In the case of a patient whose temperature had been normal for some days, a piece of bread was given to the patient by a fellow patient, causing the temperature within a few hours to reach 102·6, and which did not come down to the normal point again for six days afterwards; that was the first relapse; then a friend gives her an orange, and being ravenous and ready to use her molars on anything in the shape of solid food, she demolishes orange peel and all, with the result of prolonging the disease by about five weeks, though recovery followed eventually.

We must also consider the nursing. We may well ask ourselves the question, "What should we do without our nurses in typhoid?" They are our mainstay. They must always be on the alert for any fresh symptoms that may develop; and most willing are they to sit up day and night to watch the patient, and to administer to his wants and comforts in every way; and why is this? Not only the interest that a case of typhoid affords, in watching the progress of the disease day by day, but mainly because they know the favourable issue of the case depends, in a very great measure, upon their watchfulness and care. And they are right, for the first essential to the treatment of typhoid is to have good and sound nursing, where all orders are carried out to the letter. How much depends upon the nurse in the prevention of bed sores, by a daily application of spirit to the points of pressure on the back. We want no "Sarah Gamps" and "Betty Martins" in these enlightened days of the nineteenth century.

The next in order of importance is the dietetic treatment, and we are most careful on this point, isolating the patient as much as possible from the other patients for fear of a stray piece of bread, or other article of food finding its way to the mouth, and keeping a watchful eye on the patient's friends on visiting day; they cannot understand the patient being kept alive on milk and water only, but think it necessary for the welfare of their friend that they should smuggle in a few indigestible biscuits, or some such article of food, and are quite indignant at the refusal to allow the patient to have any.

By these means having prepared the patient for his dietetic journey, we send him along (we were going to say rejoicing, but very much afraid the patient does not think so) on milk and water, and we allow one pint of milk diluted with the same quantity of water in the twenty-four hours, given in small quantities and at short intervals during the day and night; this quantity is generally found sufficient, in fact, we have been gradually reducing the quantity; at one time two pints of milk diluted with the same quantity of water was the regulation quantity, but we find they do just as well with the smaller quantity, and there is not so much danger in setting up any intestinal complication. Ice is allowed for quenching the thirst, and this milk diet is the routine in ninety-nine cases out of a hundred. Occasionally we have a case where milk does not agree, or the patient has a natural dislike to milk, and in these cases we prescribe beef-tea, either in the shape of one of the numerous essences that abound, or, and perhaps which is the best, that made from the raw beef and then strained. A pint of this would be allowed in the twenty-four hours; this will be retained on the stomach, whereas the milk causes vomiting in these peculiar cases, for they are peculiar; because it is rarely that we find a patient who cannot take milk diluted to the extent that it is.

Another important point which will come under the dietetic treatment, is the administration of stimulants, and here we generally withhold them until there is an indication for their use, the indications being either a very high temperature, a quick, weak, or irregular pulse, toneless first sound, or a cracked, dry and furred tongue. Any of these symptoms presenting themselves, they are at once attacked with alcohol in the shape of whiskey or brandy. What we wish to impress is this, that we do not give stimulants as a routine thing, but only when symptoms present themselves, or if we can foresee them, so much the better. Small doses are commenced with, say, two or three ounces, gradually increased if necessary.

The medicinal treatment varies a good deal according to the practice of each individual physician. Some cases get no medicine at all, except perhaps a little citrate of potash with a little flavouring, other cases are treated with the so-called intestinal disinfectants, such as naphthaline, chlorine water, and minute doses of calomel. The naphthaline is given in the form of pill, or as the powder floated on milk, and four grains of this is given three times a day. The chlorine mixture is obtained very easily by pouring ʒi of the acid hydrochlor fort upon ʒi potassa chloratis and adding water to ℥xij, and one ounce of this is given three times a day. The calomel is given in doses of 1-10th or 1-12th of a grain every four hours. The great objection we find to naphthaline and chlorine water is that they are very nauseating drugs, and cause in the great majority of cases vomiting, and consequently cannot be continued for very long together. It is unfortunate that this class of drugs is so nauseating, as it seems to be a rational way of treating these cases.

With reference to the use of aperients, here also the practice varies according to the individual physician. In some cases the bowels are not allowed to be closed for more than three days, and then a dose of ol. ricini is given, a teaspoonful at first, and repeated if there is no action from the first dose. In other cases where there is constipation the bowels are not interfered with unless there is some disturbance arising from same, viz., abdominal distension or tenderness, or a rise of temperature; the ol. is then given. If there should be more than three loose motions during the day, we generally give a little astringent in the shape of opium or bismuthi subnit, gr. iv. every four hours, and if the diarrhœa is excessive a starch and opium enema.

When the patient is convalescent, that is to say when the temperature has come down to the normal point and remains so morning and evening, he is still kept on the milk and water diet for at least ten days after the temperature has become normal, for fear of setting up fresh mischief in the intestinal canal. After this the lightest food possible is allowed, a few bread crumbs in milk, or a custard, so that the way is gradually paved for more solid food.

Before concluding, we must say a few words about the complications, abdominal distension, high temperature, and hæmorrhage. With regard to abdominal distension we apply turpentine fomentations to the abdomen; the patients generally get complete relief from this application. High temperature, and by this we mean above 104 degrees F., is generally treated by sponging the patient with vinegar and water of a temperature of between 60 degrees and 70 degrees F., and continuing this sponging for a quarter of an hour or twenty minutes, or even half an hour, until the temperature has dropped two or three degrees. Great care is taken to prevent the patient getting any chill by wrapping him in blankets, and carrying out the sponging under blankets. Another method we adopt is the application of cold cloths to abdomen and chest, or wet sheets packed round the patient. The hæmorrhage we control by the application of ice to the iliac region, and by the internal administration of astringents, such as turpentine, or acetate of lead and opium in the form of pil plumbi c̄. opio, or as a mixture.

